# The frontier between cell and organelle: genome analysis of *Candidatus *Carsonella ruddii

**DOI:** 10.1186/1471-2148-7-181

**Published:** 2007-10-01

**Authors:** Javier Tamames, Rosario Gil, Amparo Latorre, Juli Peretó, Francisco J Silva, Andrés Moya

**Affiliations:** 1 Institut Cavanilles de Biodiversitat i Biologia Evolutiva, Universitat de València, Apartado Postal 22085, 46071 València, Spain; 2 Departament de Genètica, Universitat de València, Dr. Moliner, 50, 46100 Burjassot, València, Spain; 3 Departament de Bioquímica, Universitat de València, Dr. Moliner, 50, 46100 Burjassot, València, Spain

## Abstract

**Background:**

Bacterial symbioses are widespread among insects. The early establishment of such symbiotic associations has probably been one of the key factors for the evolutionary success of insects, since it may have allowed access to novel ecological niches and to new imbalanced food resources, such as plant sap or blood. Several genomes of bacterial endosymbionts of different insect species have been recently sequenced, and their biology has been extensively studied. Recently, the complete genome sequence of *Candidatus *Carsonella ruddii, considered the primary endosymbiont of the psyllid *Pachpsylla venusta*, has been published. This genome consists of a circular chromosome of 159,662 bp and has been proposed as the smallest bacterial endosymbiont genome known to date.

**Results:**

The detailed analysis of the gene content of *C. ruddii *shows that the extensive degradation of the genome is not compatible with its consideration as a mutualistic endosymbiont and, even more, as a living organism. The ability to perform most essential functions for a cell to be considered alive is heavily impaired by the lack of genes involved in DNA replication, transcription and translation. Furthermore, the shortening of genes causes, in some cases, the loss of essential domains and functional residues needed to fulfill such vital functions. In addition, at least half of the pathways towards the biosynthesis of essential amino acids, its proposed symbiotic function, are completely or partially lost.

**Conclusion:**

We propose that this strain of *C. ruddii *can be viewed as a further step towards the degeneration of the former primary endosymbiont and its transformation in a subcellular new entity between living cells and organelles. Although the transition of genes from *C. ruddii *to the host nucleus has been proposed, the amount of genes that should have been transferred to the germinal line of the insect would be so big that it would be more plausible to consider the implication of the mitochondrial machinery encoded in the insect nucleus. Furthermore, since most genes for the biosynthesis of essential amino acids have also been lost, it is likely that the host depends on another yet unidentified symbiont to complement its deficient diet.

## Background

Obligate mutualistic symbioses between insects and proteobacteria have been extensively studied in recent years [1]. The bacteria live in specialized host cells and synthesize those nutrients that are defective in the insects' restricted diets, as confirmed by genomic analyses of several endosymbionts from different insect species. Thus, *Buchnera aphidicola *and *Blochmannia *spp. provide mainly amino acids to their hosts, aphids and ants respectively [2-7], whereas *Wigglesworthia glossinidia *supplies vitamins and cofactors to the tsetse fly [8]. In the case of the sharpshooters, the genomic analysis revealed that two cohabiting endosymbionts, *Baumannia cicadellinicola *and *Sulcia muelleri *(a Bacteroidetes species) are engaged on the symbiotic relationship, providing vitamins and amino acids respectively [9].

Because they live in a protected and nutrient-rich environment, these endosymbiont genomes have experienced a reductive evolutionary process leading to smaller genome sizes than those of their free-living relatives. Nevertheless, they have retained the genes involved in the symbiotic relationship, as well as a reduced repertoire of genes necessary to maintain the three essential functions that define a living cell: maintenance, reproduction and evolution [10]. In the case of *B. aphidicola BCc*, despite its extremely reduced genome (422 kb, and only 362 protein-coding genes), it still retains a complete machinery for DNA replication, transcription and translation, and a simplified metabolic network for energy production and the synthesis of most essential amino acids needed by its aphid host. However, the loss of genes for the synthesis of tryptophan and riboflavin suggests that it cannot guarantee its host fitness, and it is plausible that a second endosymbiont could be taking on its symbiotic role [7].

*Candidatus *Carsonella ruddii, considered the primary endosymbiont of the psyllid *Pachpsylla venusta*, possess a 159,662-bp genome, with only 182 predicted open reading frames [11]. This number of genes is widely below previous proposals for minimal genomes (reviewed in [12]) and is almost half of the number of genes identified in *B. aphidicola *BCc. Such small number of genes casts doubts on the character of *C. ruddii *as a living cell. We present a detailed functional analysis of the genes retained in this small genome in order to gain new insights on the physiological role of this putative cell and the significance of the dramatic reductive process suffered by its genome.

## Results and discussion

Primary endosymbionts of insects cannot be cultured in the laboratory, making experimental analysis difficult. Therefore, the functional analysis of their genome content must rely on comparative analyses with related bacteria. However, caution must be taken when assigning a function using this approach, especially if shortened genes are identified, since some gene regions coding for specific protein domains that are related with the annotated function can be lost. In some cases, genes encoding proteins with multiple functions may lose the domain(s) responsible for some of these activities [see Additional file 2]. These circumstances might lead to the assignment of a gene to an incorrect functional category, which could lead to an erroneous determination of the set of functions a bacterium is able to perform. *C. ruddii *genes are considerably shorter than the orthologs found in other bacteria, and show extensive genetic divergence with respect to other γ-proteobacteria, mainly because their high A+T content. Therefore, the risk of over-annotation is especially high for this genome.

In order to consider that *C. ruddii *is a living organism engaged in a symbiotic relationship with its host, the genes involved in essential living functions as well as those needed for the maintenance of host fitness must be preserved. To elucidate if *C. ruddii *genome was fulfilling both conditions, we focused our analysis on the genes involved in informational processes (needed for the most essential functions for survival) and the biosynthesis of amino acids (the proposed endosymbiotic role of this biological entity).

One of the most comprehensive efforts to define the minimal core of essential genes was that presented by Gil and co-workers [13,14]. This study can be a good starting point to identify essential genes involved in informational processes that must be present in any living cell. We compared the complete set of genes of the minimal genome proposed by Gil *et al*. [13] with the gene repertoire of *C. ruddii *after the reannotation analysis performed as described in Methods. The results of the analysis clearly show that the *C. ruddii *gene complement is not sufficient to replicate, transcribe and synthesize proteins (Table 1), thus questioning its consideration as an independent living entity. It is worth mentioning that only 29 genes remain as orphan after our genome reannotation [see Additional file 1]. However, even if we could assign a function to them, many essential functions will still be lacking. Regarding DNA maintenance and replication, no genes for histone-like and single stranded-binding proteins (involved in the structural maintenance and protection of the chromosomal DNA) are found on the genome; replication initiation must be dependent on RecA, since no recruiting proteins are present [15]; the complete DNA replisome is only represented by the two essential core subunits of the DNA polymerase, since the putative genes for the helicase and primase (two enzymes needed for the initiation of the replication, by separating the strands of the double helix and priming the single strand that is going to be replicated with an RNA primer) are highly degraded [see Additional file 2]. Furthermore, no genes for gyrase (needed to relax the positive supercoils generated by the replication process), ligase (essential for joining the DNA fragments in the lagging strand), and RNAse HI (needed to eliminate the RNA primer) have been found. The transcription machinery is limited to the core subunits of RNA polymerase, since the gene for the proposed sigma factor subunit has lost most of the sequence, including the residues involved in the promoter recognition, and no other transcription factors have been annotated. Finally, the translation machinery is highly reduced. It contains the minimal set of RNA genes required for protein synthesis, including the three rRNA genes and 28 tRNAs genes, enough for decoding the 61 codons of the genetic code, although the gene *mesJ*, coding for the tRNA^Ile^-lysidine synthethase required for the maturation of tRNA^Ile ^(CAT), is missing. Moreover, at least 9 aminoacyl-tRNA synthetases and 15 out of 50 proposed essential ribosomal protein components are missing or degraded, questioning the capacity of *C. ruddii *to build functional ribosomes. Seven previously unnanotated ORFs could in fact be some remnants of ribosomal protein genes, based on its genome position and considering some degree of synteny with other completely sequenced γ-proteobacteria, but only three of them (*rplQ*, *rpsE*, and *rpsO*) retain enough homology to be annotated as such. Finally, most ribosome maturation proteins and several essential translation factors (such as the elongation factors P and Ts) are also missing from this genome.

**Table 1:** Essential genes involved in informational processes that are missing or degraded in *C. ruddii *genome.

**Functional category**	**Product**	**Gene**	**Status**	**Comments**
Chromosome-associated protein	DNA-binding protein	hupA*	Absent	
DNA replication	replicative DNA helicase	dnaB	Degraded	Helicase domain is very degraded
DNA replication	DNA primase	dnaG	Degraded	Helicase-binding domain and part of DNA-binding domain are missing
DNA replication	DNA polymerase III, b subunit	dnaN	Absent	
DNA replication	DNA polymerase III, d subunit	holA	Absent	
DNA replication	DNA polymerase III, d' subunit	holB	Absent	
DNA replication	DNA polymerase III, g and t subunits	dnaX	Absent	
DNA replication	DNA gyrase, A subunit	gyrA	Absent	
DNA replication	DNA gyrase, B subunit	gyrB	Absent	
DNA replication	DNA ligase	lig	Absent	
DNA replication	single-strand DNA-binding protein	ssb	Absent	
DNA repair, restriction, and modification	exonuclease	polA*	Absent	
DNA repair, restriction, and modification	uracil-DNA glycosylase	ung	Absent	
Transcription	RNA polymerase major sigma factor	rpoD	Degraded	Three out of six domains of the protein have been lost. Other two are very degraded, including those responsible to bind both to -10 promoter region and RNA polymerase.
Transcription	ATP-dependent RNA helicase	deaD	Absent	
Transcription	transcription elongation factor	greA	Absent	
Transcription	transcription pausing; L factor	nusA	Absent	
Transcription	transcription antitermination protein	nusG	Absent	
Aminoacyl-tRNA synthetase	arginyl-tRNA synthetase	argS	Absent	
Aminoacyl-tRNA synthetase	asparaginyl-tRNA synthetase	asnS	Absent	
Aminoacyl-tRNA synthetase	cysteinyl-tRNA synthetase	cysS	Absent	
Aminoacyl-tRNA synthetase	glycyl-tRNA synthetase, β subunit	glyS	Absent	
Aminoacyl-tRNA synthetase	histidyl-tRNA synthetase	hisS	Absent	
Aminoacyl-tRNA synthetase	phenylalanyl-tRNA synthetase, a subunit	pheS	Degraded	Aminoacyl tRNA synthetase class II N-terminal domain, and part of tRNA synthetases class II core domain are missing
Aminoacyl-tRNA synthetase	phenylalanyl-tRNA synthetase, b subunit	pheT	Absent	
Aminoacyl-tRNA synthetase	prolyl-tRNA synthetase	proS	Absent	
Aminoacyl-tRNA synthetase	threonyl-tRNA synthetase	thrS	Absent	
Aminoacyl-tRNA synthetase	valyl-tRNA synthetase	valS	Degraded	Most of the anticodon domain is missing. Mutations in functional residues
tRNA maduration and modification	tRNA(Ile)-lysidine synthetase	mesJ	Absent	
tRNA maduration and modification	peptidyl-tRNA hydrolase	pth	Absent	
tRNA maduration and modification	protein component of ribonuclease P	rnpA	Absent	
Ribosomal protein	50S ribosomal protein L3	rplC	Degraded	Loss of first half of the protein
Ribosomal protein	50S ribosomal protein L9	rplI	Absent	
Ribosomal protein	50S ribosomal protein L13	rplM	Degraded	Not matching family profile. Well conserved zones are mutated
Ribosomal protein	50S ribosomal protein L19	rplS	Absent	
Ribosomal protein	50S ribosomal protein L23	rplW	Absent	
Ribosomal protein	50S ribosomal protein L24	rplX	Absent	
Ribosomal protein	50S ribosomal protein L29	rpmC	Absent	
Ribosomal protein	50S ribosomal protein L34	rpmH	Absent	
Ribosomal protein	50S ribosomal protein L35	rpmI	Absent	
Ribosomal protein	30S ribosomal protein S6	rpsF	Absent	
Ribosomal protein	30S ribosomal protein S18	rpsR	Absent	
Ribosomal protein	30S ribosomal protein S20	rpsT	Absent	
Ribosome function	GTP-binding protein	era	Absent	
Ribosome function	ribosomal methytransferase	cspR	Absent	
Ribosome function	dimethyladenosine transferase	ksgA	Absent	
Ribosome function	ribosome-binding factor A	rbfA	Absent	
Ribosome function	GTP-binding protein	ychF	Absent	
Ribosome function	GTP-binding protein	engA	Absent	
Ribosome function	GTP-binding protein, Obg family	obg (yhbZ)	Degraded	GTP binding sites are lost
Translation factors	elongation factor P	efp	Absent	
Translation factors	ribosome recycling factor	frr	Degraded	Mutations in residues interacting with the ribosome, known to abolish functionality
Translation factors	N5-glutamine methyltransferase	hemK	Absent	
Translation factors	GTP-binding elongation factor	lepA	Absent	
Translation factors	tmRNA-binding protein	smpB	Degraded	Missing some β strands important for β-barrel structure. Mutations in zones interacting with RNA.
Translation factors	elongation factor Ts.	tsf	Degraded	Mutations in D80-F81, interacting with Ef-Tu, known to abolish functionality
RNA degradation	polyribonucleotide nucleotidyltransferase	pnp	Absent	
RNA degradation	ribonuclease III	rnc	Absent	

* The gene name corresponds to the one selected in the proposed minimal genome [13]. Although there are other genes for the same function in γ-proteobacteria, none of them is present in *C. ruddii*.

As it lacks most genes for DNA replication, transcription and translation, this biological entity might be dependent on an external source for these functions. Although the transition of genes from *C. ruddii *to the host nucleus has been proposed [11], it should be taken into account that the ancestor of the psyllids infected by the ancestor of *C. ruddi *was already a complex multicellular organism, so that all transferred genes must have been acquired by the germinal cell line. The amount of genes that should have been transferred is so big that it would be more plausible to consider that this intracellular entity takes advantage of some nuclear genes involved in the mitochondrial activity. The dual target of proteins encoded by the eukaryotic nucleus between different organelles (mitochondria and plastids) has already been described in plant cells, especially regarding genes involved in informational processes [16,17].

In addition to the essential functions that define life, an endosymbiont must provide its host all essential complements to its nutritionally deficient diet. Psyllids feed on phloem sap, rich in sugars but relatively poor in nitrogenated compounds, especially essential amino acids. Since it has been proposed that the role of *C. ruddii*, similarly to *B. aphidicola*, is providing those amino acids to its host [18], we looked for the maintenance of all pathways involved in essential amino acids biosynthesis [see Additional file 3] [19,20]. The analysis revealed that the pathways for the synthesis of histidine, phenylalanine and tryptophan are absent. Moreover, although the threonine biosynthetic pathway is complete, *thrB *is probably not functional, and thus an activity as yet unidentified should supply such function. The same case happens in *Candidatus *Ruthia magnifica, an autotrophic endosymbiotic with a complete metabolic network in which the *thrB *gene is absent in the context of an otherwise complete threonine biosynthetic pathway [21]. This limited provision of amino acids is not enough to sustain the requirements of its host. The loss of essential endosymbiotic functions has also been detected in other insect-bacteria associations but, in such cases, a second symbiont appears to be complementing the insect diet [7,9]. Surprisingly, although secondary symbionts have been found in other psyllids, no other symbionts were detected in *Pachpsylla venusta *[11,18]. Nevertheless, such statement is based on a single work, where only bacterial symbionts were searched by PCR amplification of 16S-23S rDNA [18], and the absence of contaminating sequences during the sequencing project, which was performed on DNA purified from bacteriocytes [11]. Therefore, a problem on a specific amplification reaction cannot be discarded, and an extracellular and/or eukaryotic symbiont would not have been detected by these analyses [22,23]. If another partner is detected in this symbiotic association, the possibility that *C. ruddii *is the remnant of an ancient endosymbiont that is being driven towards its extinction and replacement by the new symbiont, as it has already been proposed in other insects [7,22,24], cannot be ruled out.

## Conclusion

A careful functional analysis of the gene repertoire of *C. ruddii *reveals that the extensive degradation of its genome is not compatible with its consideration as a mutualistic endosymbiont and, even more, as a living organism. Although *C. ruddii *is defined as a psyllid primary endosymbiont, the genes for the biosynthesis of three essential amino acids have been completely lost. This observation raises doubts about both the role of *C. ruddii *in the symbiotic relationship with its host and the absence of a secondary symbiont capable to provide the rest of necessary nutrients to complement the unbalanced insect diet. Although a bacterial symbiont has not been found in the psyllid *Pachpsylla venusta*, further studies need to be conducted in order to detect the possibility of a second, maybe eukaryotic, symbiont.

We propose that this strain of *C. ruddii *can be viewed as a further step towards the degeneration of the former primary endosymbiont, and its transformation in a subcellular new entity between living cells and organelles, which probably would take advantage of mitochondrial functions encoded by the nucleus, especially for basic informational processes needed for maintenance and multiplication. If confirmed, this would be the first example of such a scenario in animal cells.

## Methods

In order to confirm the presence and functionality of all ORFs identified in the original report and search for additional functions, the complete genome sequence of *C. ruddii *(accession number AP009180) was re-analyzed. This is particularly relevant in this case, as 46 of the putative genes (25% of the genome) are annotated as hypothetical ORFs in the original report. ORFs with putative functions were obtained from the original annotation of the genome and complemented with Glimmer predictions [25]. This set of putative ORFs was checked by homology searches using BLASTX and the latest version of GenBank database. All hits with e-values above 1e-02 were disregarded. Whenever a clear homology was found, the putative ORF was translated, and the protein sequences were aligned using ClustalW [26] with the corresponding translated orthologous genes found in *E. coli *and all sequenced *B. aphidicola *genomes (accession numbers U00096.2, BA000003, AE013218, AE016826 and CP000263). Many proteins were found to be rather degraded, presenting numerous deletions and a high number of amino acid changes. In order to confirm the maintenance of the original function, we looked for the presence of the domains and active residues (if known) responsible for functionality, using information in Uniprot, Pfam, EcoCyc, and EcoGene databases [27-30]. The secondary structures of the resulting proteins and their orthologs were also compared, to exclude major structural changes that could impede function.

Looking for possible essential functions that might have been missed on the original annotation, the genes responsible for these functions in γ-proteobacteria were retrieved, and used to build Hidden Markov Models and perform searches in the *C. ruddii *genome with HMMER [31]. Manual inspection of the results may allow detecting subtle homologies that could have been missed in the BLASTX search. In addition, positional information was taken into account to identify genes that maintain synteny with other completely sequenced γ-proteobacteria genomes. All combined strategies allowed the annotation of 17 previously considered orphan genes, six of which appear to be functional. In addition, 9 previously annotated genes were considered unfunctional [see Additional file 1]. Therefore, there are only 29 ORFs in the *C. ruddii *genome that remain annotated as hypothetical proteins, while 20 ORFs can be traced back to ancestral functional genes, although the changes in the sequence indicate that they probably are non-functional.

Ribosomal RNA genes were confirmed with BLASTN. Anticodons for tRNA genes were identified with tRNAscan-SE [32]. Discrimination of tRNA genes with anticodon CAT was performed with the program TFAM [33] using the tRNA profiles of initiator tRNA^Met^, elongator tRNA^Met ^and tRNA^Ile^(CAT) [34].

Metabolic pathways were reconstructed using KEGG [35], and enzyme characteristics were checked in BRENDA [36].

## Competing interests

The author(s) declares that there are no competing interests.

## Authors' contributions

JT and RG conceived the study and carried out the manual curation of the gene functional assignments. JT performed the bioinformatics analyses. RG and JP carried out the metabolic analyses. FJS performed the tRNA studies. JT, RG and AL drafted the manuscript. AM participated in design and coordination and helped to draft the manuscript. All authors participated in the discussion of the biological implications of the results, read and approved the final manuscript.

## Supplementary Material

Additional file 2 Alignment of CRP_058 of *C.rudii *(*dnaG*, DNA primase) and its orthologs in *E.coli *(eco), *B. aphidicola *from the aphids *Acyrtosiphon pisum *(buc), *Baizongia pistacea *(bab), *Schizaphis graminum *(bas), and *Cinara cedri *(bcc). The protein in *C. ruddii *is extensively degraded, and has partial or completely lost two domains that are critical for functionality: The Zn-finger domain (shadowed in pink), needed for binding to DNA, and the DnaG-DnaB binding domain (shadowed in blue), needed for interacting with helicase DnaB (also very degraded). Click here for file

Additional file 1 Table summarizing the results of the reannotation of the *Carsonella rudii*'s genome, as described in the main text. Click here for file

Additional file 3 Schematic representation of amino acid biosynthetic pathways in *E. coli *(blue arrows), *B. aphidicola *BAp (green arrows), and *C. rudii *(red arrows). Only incomplete pathways in *C. rudii *are shown. Hatched arrows in *C. rudii *indicate genes that are present in its genome but are greatly degraded. These genes probably have lost their original functionality. Open arrows show activities that have not been found in the genome but could be encoded by some other gene. The metabolites shown in magenta must be provided by the host. Click here for file

## References

[B1] Baumann P (2005). Biology bacteriocyte-associated endosymbionts of plant sap-sucking insects. Annu Rev Microbiol.

[B2] Shigenobu S, Watanabe H, Hattori M, Sakaki Y, Ishikawa H (2000). Genome sequence of the endocellular bacterial symbiont of aphids Buchnera sp. APS. Nature.

[B3] Tamas I, Klasson L, Canback B, Naslund AK, Eriksson AS, Wernegreen JJ, Sandstrom JP, Moran NA, Andersson SG (2002). 50 million years of genomic stasis in endosymbiotic bacteria. Science.

[B4] van Ham RC, Kamerbeek J, Palacios C, Rausell C, Abascal F, Bastolla U, Fernandez JM, Jimenez L, Postigo M, Silva FJ, Tamames J, Viguera E, Latorre A, Valencia A, Moran F, Moya A (2003). Reductive genome evolution in Buchnera aphidicola. Proc Natl Acad Sci U S A.

[B5] Degnan PH, Lazarus AB, Wernegreen JJ (2005). Genome sequence of Blochmannia pennsylvanicus indicates parallel evolutionary trends among bacterial mutualists of insects. Genome Res.

[B6] Gil R, Silva FJ, Zientz E, Delmotte F, Gonzalez-Candelas F, Latorre A, Rausell C, Kamerbeek J, Gadau J, Holldobler B, van Ham RC, Gross R, Moya A (2003). The genome sequence of Blochmannia floridanus: comparative analysis of reduced genomes. Proc Natl Acad Sci U S A.

[B7] Perez-Brocal V, Gil R, Ramos S, Lamelas A, Postigo M, Michelena JM, Silva FJ, Moya A, Latorre A (2006). A small microbial genome: the end of a long symbiotic relationship?. Science.

[B8] Akman L, Yamashita A, Watanabe H, Oshima K, Shiba T, Hattori M, Aksoy S (2002). Genome sequence of the endocellular obligate symbiont of tsetse flies, Wigglesworthia glossinidia. Nat Genet.

[B9] Wu D, Daugherty SC, Van Aken SE, Pai GH, Watkins KL, Khouri H, Tallon LJ, Zaborsky JM, Dunbar HE, Tran PL, Moran NA, Eisen JA (2006). Metabolic complementarity and genomics of the dual bacterial symbiosis of sharpshooters. PLoS Biol.

[B10] Luisi PL, Oberholzer T, Lazcano A (2002). The notion of a DNA minimal cell: a general discourse and some gidelines for an experimental approach. Helv Chim Acta.

[B11] Nakabachi A, Yamashita A, Toh H, Ishikawa H, Dunbar HE, Moran NA, Hattori M (2006). The 160-kilobase genome of the bacterial endosymbiont Carsonella. Science.

[B12] Gil R, Pérez-Brocal V, Latorre A, Moya A, Logan NA, Lappin-Scott HM, Oyston CF (2006). Minimal genomes required for life. Prokaryotic diversity Mecanisms and significance.

[B13] Gil R, Silva FJ, Pereto J, Moya A (2004). Determination of the core of a minimal bacterial gene set. Microbiol Mol Biol Rev.

[B14] Glass J Faculty of 1000 Biology. http://www.f1000biology.com/article/id/1021418/evaluation.

[B15] Hong X, Cadwell GW, Kogoma T (1996). Activation of stable DNA replication in rapidly growing Escherichia coli at the time of entry to stationary phase. Molecular Microbiology.

[B16] Duchene AM, Giritch A, Hoffmann B, Cognat V, Lancelin D, Peeters NM, Zaepfel M, Marechal-Drouard L, Small ID (2005). Dual targeting is the rule for organellar aminoacyl-tRNA synthetases in Arabidopsis thaliana. Proc Natl Acad Sci U S A.

[B17] Mackenzie SA (2005). Plant organellar protein targeting: a traffic plan still under construction. Trends Cell Biol.

[B18] Thao ML, Moran NA, Abbot P, Brennan EB, Burckhardt DH, Baumann P (2000). Cospeciation of psyllids and their primary prokaryotic endosymbionts. Appl Environ Microbiol.

[B19] Douglas AE (2006). Phloem-sap feeding by animals: problems and solutions. J Exp Bot.

[B20] Zientz E, Dandekar T, Gross R (2004). Metabolic Interdependence of Obligate Intracellular Bacteria and Their Insect Hosts. Microbiol Mol Biol Rev.

[B21] Newton IL, Woyke T, Auchtung TA, Dilly GF, Dutton RJ, Fisher MC, Fontanez KM, Lau E, Stewart FJ, Richardson PM, Barry KW, Saunders E, Detter JC, Wu D, Eisen JA, Cavanaugh CM (2007). The Calyptogena magnifica chemoautotrophic symbiont genome. Science.

[B22] Fukatsu T, Ishikawa H (1992). A novel eukaryotic extracellular symbiont in an aphid, Astegopterys styraci (Homoptera, Aphidinae, Hormaphidinae). J Insect Physiol.

[B23] Hosokawa T, Kikuchi Y, Nikoh N, Shimada M, Fukatsu T (2006). Strict host-symbiont cospeciation and reductive genome evolution in insect gut bacteria. PLoS Biol.

[B24] Lefevre C, Charles H, Vallier A, Delobel B, Farrell B, Heddi A (2004). Endosymbiont Phylogenesis in the Dryophthoridae Weevils: Evidence for Bacterial Replacement. Mol Biol Evol.

[B25] Delcher AL, Harmon D, Kasif S, White O, Salzberg SL (1999). Improved microbial gene identification with GLIMMER. Nucl Acids Res.

[B26] Thompson JD, Higgins DG, Gibson TJ (1994). CLUSTAL W: improving the sensitivity of progressive multiple sequence alignment through sequence weighting, position-specific gap penalties and weight matrix choice. Nucleic Acids Res.

[B27] EcoCyc: Encyclopedia of Escherichia coli K-12 Genes and Metabolism. http://ecocyc.org/.

[B28] EcoGene: The EcoGene Database of Escherichia coli Sequence and Function. http://ecogene.org/.

[B29] Pfam: Protein families database. http://www.sanger.ac.uk/Software/Pfam/.

[B30] Uniprot, the universal protein resource. http://www.ebi.uniprot.org/index.shtml.

[B31] Eddy SR (1998). Profile hidden Markov models. Bioinformatics.

[B32] Lowe TM, Eddy SR (1997). tRNAscan-SE: a program for improved detection of transfer RNA genes in genomic sequence. Nucleic Acids Res.

[B33] Ardell DH, Andersson SG (2006). TFAM detects co-evolution of tRNA identity rules with lateral transfer of histidyl-tRNA synthetase. Nucleic Acids Res.

[B34] Silva FJ, Belda E, Talens SE (2006). Differential annotation of tRNA genes with anticodon CAT in bacterial genomes. Nucleic Acids Res.

[B35] Kanehisa M, Goto S (2000). KEGG: Kyoto Encyclopedia of Genes and Genomes. Nucl Acids Res.

[B36] Schomburg I, Chang A, Schomburg D (2002). BRENDA, enzyme data and metabolic information. Nucl Acids Res.

