# Supplementary figures and images for: The frontier between cell and organelle: genome analysis of Candidatus Carsonella ruddii
Source: BMC Evol Biol. 2007 Oct 1;7:181. doi: 10.1186/1471-2148-7-181 (PMC2175510; doi:10.1186/1471-2148-7-181)

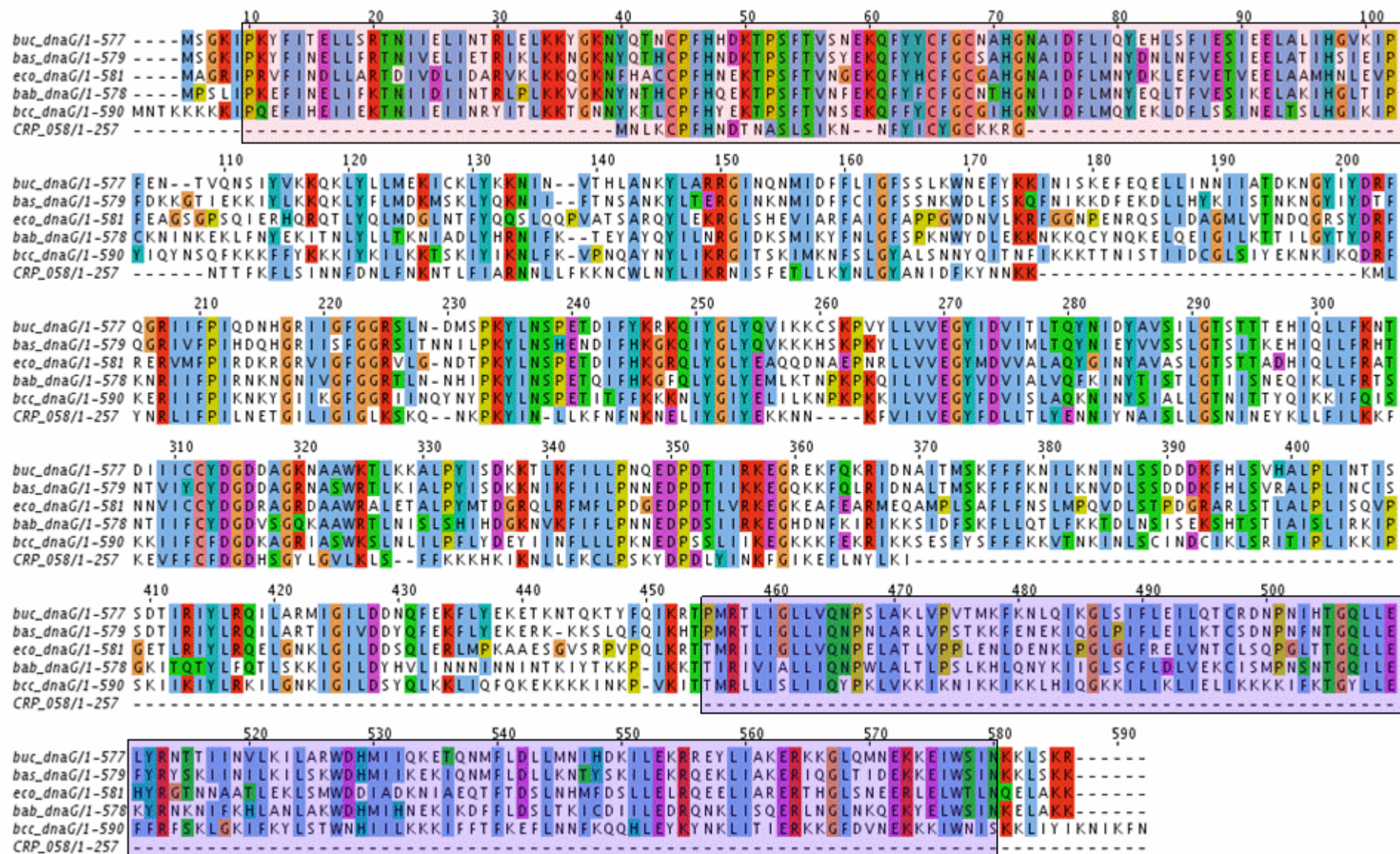

Figure S1

Supplement: Additional file 2 — Alignment of CRP_058 of C.rudii (dnaG, DNA primase) and its orthologs in E.coli (eco), B. aphidicola from the aphids Acyrtosiphon pisum (buc), Baizongia pistacea (bab), Schizaphis graminum (bas), and Cinara cedri (bcc). The protein in C. ruddii is extensively degraded, and has partial or completely lost two domains that are critical for functionality: The Zn-finger domain (shadowed in pink), needed for binding to DNA, and the DnaG-DnaB binding domain (shadowed in blue), needed for interacting with helicase DnaB (also very degraded). [file 1471-2148-7-181-S2.pdf]
