# Supplementary material for: The frontier between cell and organelle: genome analysis of Candidatus Carsonella ruddii
Source: BMC Evol Biol. 2007 Oct 1;7:181. doi: 10.1186/1471-2148-7-181 (PMC2175510; doi:10.1186/1471-2148-7-181)
Supplement: Additional file 1 — Table summarizing the results of the reannotation of the Carsonella rudii's genome, as described in the main text. [file 1471-2148-7-181-S1.doc]

**Supplementary table S1.** Reannotated genome of *Carsonella ruddii*

| **Product Name** | **Gene name*^a^*** | ***E. coli*** | **Original annotation** | **Comments** | **Functional category** |
| --- | --- | --- | --- | --- | --- |
| tRNA modification GTPase | **mnmE (thdF)** | b3706 |  |  | tRNA maturation and modification |
| glucose inhibited division protein A | **mnmG (gidA)** | b3741 |  |  | tRNA maturation and modification |
| F0F1-type ATP synthase A subunit | **atpB** | b3738 |  |  | Energy metabolism |
| F0F1-type ATP synthase C subunit | **atpE** | b3737 |  |  | Energy metabolism |
| F0F1-type ATP synthase B subunit | **atpF** | b3736 | putative F0F1-type ATP synthase B subunit |  | Energy metabolism |
| putative unfunctional F0F1-type ATP synthase delta subunit | **atpH** | b3735 | hypothetical protein CRP_006 | Identified by syntheny. | Energy metabolism |
| F0F1-type ATP synthase alpha subunit | **atpA** | b3734 |  |  | Energy metabolism |
| F0F1-type ATP synthase gamma subunit | **atpG** | b3733 |  |  | Energy metabolism |
| F0F1-type ATP synthase beta subunit | **atpD** | b3732 |  |  | Energy metabolism |
| putative unfunctional F0F1-type ATP synthase epsilon subunit | **atpC** | b3731 | hypothetical protein CRP_010 | Identified by homology. | Energy metabolism |
| ornithine carbamoyltransferase | **argF** | b0273 |  |  | Amino acid metabolism |
| 3-dehydroquinate dehydratase | **aroD** | b1693 |  |  | Amino acid metabolism |
| transketolase | **tktB** | b2465 |  |  | Carbohydrate metabolism |
| hypothetical protein CRP_014 |  |  |  |  |  |
| putative unfunctional 5,10-methylenetetrahydrofolate reductase | **metF** | b3941 | 5,10-methylenetetrahydrofolate reductase | Flavin-binding residues are lost (Ala 177, Asn 168, Asp 165) [1]. | Cofactors metabolism |
| hypothetical protein CRP_016 |  |  |  |  |  |
| succinyl-diaminopimelate desuccinylase | **dapE** | b2472 |  |  | Amino acid metabolism |
| tetrahydrodipicolinate N-succinyltransferase | **dapD** | b0166 |  |  | Amino acid metabolism |
| methionine aminopeptidase | **map** | b0168 |  |  | Protein processing, folding and secretion |
| ribosomal protein S2 | **rpsB** | b0169 |  |  | Ribosomal proteins |
| putative unfunctional elongation factor EF-Ts | **tsf** | b0170 | hypothetical protein CRP_021 | Identified by homology. Important residues for the interaction with EF-Tu (Asp80, Phe81) and Mg2+ are lost [2]. | Translation factors |
| putative unfunctional ribosome recycling factor | **frr** | b0172 | hypothetical protein CRP_022 | Identified by homology. Residues interacting with the ribosome are poorly conserved [3]. | Translation factors |
| DNA polymerase III alpha subunit | **dnaE** | b0184 |  |  | DNA replication |
| hypothetical protein CRP_024 |  |  |  |  |  |
| 5-methyltetrahydropteroyltriglutamate-- homocysteine S-methyltransferase | **metE** | b3829 |  |  | Amino acid metabolism |
| ribosomal protein L31 | **rpmE** | b3936 |  |  | Ribosomal proteins |
| putative unfunctional shikimate kinase I | **aroK** | b3390 | hypothetical protein CRP_027 | Identified by homology. Catalytic site not completely conserved. | Amino acid metabolism |
| 3-dehydroquinate synthase | **aroB** | b3389 |  |  | Amino acid metabolism |
| dihydroxy-acid dehydratase | **ilvD** | b3771 |  |  | Amino acid metabolism |
| hypothetical protein CRP_030 |  |  |  |  |  |
| serine hydroxymethyltransferase | **glyA** | b2551 |  |  | Cofactors metabolism |
| putative ABC transporter permease component |  |  |  |  |  |
| putative ABC transporter ATP-binding component |  |  |  |  |  |
| hypothetical protein CRP_034 |  |  |  |  |  |
| putative selenocysteine lyase | **iscS (yfhO)** | b2530 | selenocysteine lyase | Catalytic site badly conserved. | tRNA maduration and modification |
| putative tRNA(5-methylaminomethyl-2- thiouridylate) methyltransferase | **mnmA (trmU)** | b1133 |  |  | tRNA maduration and modification |
| translation initiation factor IF-1 | **infA** | b0884 |  |  | Translation factors |
| putative thioredoxin | **trxA** | b3781 |  |  | Energy metabolism |
| 6-phosphogluconate dehydrogenase | **gnd** | b2029 |  |  | Carbohydrate metabolism |
| hypothetical protein CRP_040 |  |  |  |  |  |
| putative peptide chain release factor A | **prfA** | b1211 |  |  | Translation factors |
| aspartyl/glutamyl-tRNA amidotransferase B subunit | **gatB** |  |  |  |  |
| aspartyl/glutamyl-tRNA amidotransferase A subunit | **gatA** |  |  |  |  |
| hypothetical protein CRP_044 |  |  |  |  |  |
| putative unfunctional ribosomal protein L13 | **rplM** | b3231 | ribosomal protein L13 | Well conserved regions in this family are lost | Ribosomal proteins |
| ribosomal protein S9 | **rpsI** | b3230 |  |  | Ribosomal proteins |
| hypothetical protein CRP_047 |  |  |  |  |  |
| hypothetical protein CRP_048 |  |  |  |  |  |
| 3-phosphoshikimate 1-carboxyvinyltransferase | **aroA** | b0908 |  |  | Amino acid metabolism |
| putative ribosomal protein S1 | **rpsA** | b0911 |  |  | Ribosomal proteins |
| chaperonin GroEL | **groEL** | b4143 |  |  | Protein processing, folding and secretion |
| chaperonin GroES | **groES** | b4142 |  |  | Protein processing, folding and secretion |
| hypothetical protein CRP_053 |  |  |  |  |  |
| hypothetical protein CRP_054 |  |  |  |  |  |
| putative unfunctional replicative DNA helicase | **dnaB** | b4052 | putative replicative DNA helicase | Helicase domain has been lost | DNA replication |
| hypothetical protein CRP_056 |  |  |  |  |  |
| hypothetical protein CRP_057 |  |  |  |  |  |
| putative unfunctional DNA primase | **dnaG** | b3066 | putative DNA primase | More than half the protein has been lost, including the helicase-binding domain and most of the DNA-binding domain. | DNA replication |
| putative unfunctional RNA polymerase sigma factor rpoD | **rpoD** | b3067 | putative RNA polymerase sigma factor rpoD | Three out of six domains of the protein have been lost. Other two are very degraded, including these involved in binding the -10 promoter region and the RNA polymerase. | Transcription |
| putative unfunctional tmRNA-binding protein | **smpB** | b2620 | hypothetical protein CRP_060 | Identified by homology. Missing some b strands important for b-barrel structure. Mutations in zones interacting with RNA [4]. | Translation factors |
| chaperone protein GrpE | **grpE** | b2614 |  |  | Protein processing, folding and secretion |
| chaperone protein DnaK | **dnaK** | b0014 |  |  | Protein processing, folding and secretion |
| hypothetical protein CRP_063 |  |  |  |  |  |
| dihydrodipicolinate reductase | **dapB** | b0031 |  |  | Amino acid metabolism |
| truncated carbamoylphosphate synthase small subunit | **carA-1** | b0032 |  |  | Amino acid metabolism |
| truncated carbamoylphosphate synthase small subunit | **carA-2** | b0032 |  |  |  |
| carbamoylphosphate synthase large subunit | **carB** | b0033 |  |  | Amino acid metabolism |
| putative translation initiation factor IF-2 | **infB** | b3168 |  |  | Translation factors |
| ribosomal protein S15 | **rpsO** | b3165 | hypothetical protein CRP_069 | Identified by homology | Ribosomal proteins |
| tyrosyl-tRNA synthetase | **tyrS** | b1637 |  |  | Aminoacyl-tRNA synthetases |
| glutaminyl-tRNA synthetase | **glnS** | b0680 |  |  | Aminoacyl-tRNA synthetases |
| ATP-dependent Clp protease proteolytic subunit | **clpP** | b0437 |  |  | Protein processing, folding and secretion |
| ATP-dependent Clp protease ATP-binding subunit | **clpX** | b0438 |  |  | Protein processing, folding and secretion |
| putative endonuclease | **cls***^b^* | b2159 | hypothetical protein CRP_074 | Matches the cardiolipin synthase PLDc domain (Phospholipase D), known to be present in several endonucleases [5]. | DNA repair, restriction, and modification |
| aspartyl-tRNA synthetase | **aspS** | b1866 |  |  | Aminoacyl-tRNA synthetases |
| ribulose-phosphate 3-epimerase | **rpe** | b3386 |  |  | Carbohydrate metabolism |
| delta-1-pyrroline-5-carboxylate dehydrogenase | **putA** | b1014 |  |  |  |
| 2-isopropylmalate synthase | **leuA** | b0074 |  |  | Amino acid metabolism |
| chorismate synthase | **aroC** | b2329 |  |  | Amino acid metabolism |
| 3-isopropylmalate dehydratase large subunit | **leuC** | b0072 |  |  | Amino acid metabolism |
| 3-isopropylmalate dehydratase small subunit | **leuD** | b0071 |  |  | Amino acid metabolism |
| 3-isopropylmalate dehydrogenase | **leuB** | b0073 |  |  | Amino acid metabolism |
| aspartate-semialdehyde dehydrogenase | **asd** | b3433 |  |  | Amino acid metabolism |
| seryl-tRNA synthetase | **serS** | b0893 |  |  | Aminoacyl-tRNA synthetases |
| hypothetical protein CRP_085 |  |  |  |  |  |
| hypothetical protein CRP_086 |  |  |  |  |  |
| DNA polymerase III epsilon subunit | **dnaQ** | b0215 |  |  | DNA replication |
| hypothetical protein CRP_088 |  |  |  |  |  |
| putative unfunctional phenylalanyl-tRNA synthetase alpha subunit | **pheS** | b1714 | putative phenylalanyl-tRNA synthetase alpha subunit | Lacks the N-terminal domain plus most of the C-terminal region. | Aminoacyl-tRNA synthetases |
| ribosomal protein L20 | **rplT** | b1716 |  |  | Ribosomal proteins |
| translation initiation factor IF-3 | **infC** | b1718 |  |  | Translation factors |
| superoxide dismutase | **sodA** | b3908 |  |  | Detoxification |
| hypothetical protein CRP_093 |  |  |  |  |  |
| 3-deoxy-7-phosphoheptulonate synthase | **aroH** | b1704 |  |  | Amino acid metabolism |
| ribosomal protein L32 | **rpmF** | b1089 | hypothetical protein CRP_095 | Identified by homology. | Ribosomal proteins |
| isopentenyl-adenosine tRNA methylthiolase | **yleA** | b0661 | hypothetical protein CRP_096 | Identified by homology. | tRNA maduration and modification |
| hypothetical protein CRP_097 |  |  |  |  |  |
| leucyl-tRNA synthetase | **leuS** | b0642 |  |  | Aminoacyl-tRNA synthetases |
| succinyl-CoA synthetase alpha subunit |  |  |  |  |  |
| succinyl-CoA synthetase beta subunit |  |  |  |  |  |
| putative glutamyl-tRNA synthetase | **gltX** | b2400 |  |  | Aminoacyl-tRNA synthetases |
| methionyl-tRNA synthetase | **metG** | b2114 |  |  | Aminoacyl-tRNA synthetases |
| hypothetical protein CRP_103 |  |  |  |  |  |
| argininosuccinate synthase | **argG** |  |  |  | Amino acid metabolism |
| dihydrodipicolinate synthase | **dapA** | b2478 |  |  | Amino acid metabolism |
| hypothetical protein CRP_106 |  |  |  |  |  |
| malate:quinone oxidoreductase | **mqo** |  |  |  |  |
| aspartokinase | **lysC** | b4024 |  |  | Amino acid metabolism |
| putative alanyl-tRNA synthetase | **alaS** | b2697 |  |  | Aminoacyl-tRNA synthetases |
| RecA recombinase | **recA** |  |  |  |  |
| putative unfunctional valyl-tRNA synthetase | **valS** | b4258 | valyl-tRNA synthetase | Most of the anticodon-domain has been lost, together with 40% of the well conserved sequence in this family | Aminoacyl-tRNA synthetases |
| alkyl hydroperoxide reductase | **ahpC** | b0605 |  |  | Detoxification |
| transaldolase | **talA** | b2464 |  |  | Carbohydrate metabolism |
| tryptophanyl-tRNA synthetase | **trpS** | b3384 |  |  | Aminoacyl-tRNA synthetases |
| ribosomal protein L33 | **rpmG** | b3636 |  |  | Ribosomal proteins |
| ribosomal protein L28 | **rpmB** | b3637 |  |  | Ribosomal proteins |
| hypothetical protein CRP_117 |  |  |  |  |  |
| lysyl-tRNA synthetase | **lysS** | b2890 |  |  | Aminoacyl-tRNA synthetases |
| peptide chain release factor B | **prfB** | b2891 |  |  | Translation factors |
| threonine synthase | **thrC** | b0004 |  |  | Amino acid metabolism |
| homoserine dehydrogenase | **thrA** | b0002 |  |  | Amino acid metabolism |
| hypothetical protein CRP_122 |  |  |  |  |  |
| hypothetical protein CRP_123 |  |  |  |  |  |
| ribosomal protein S16 | **rpsP** | b2609 |  |  | Ribosomal proteins |
| ketol-acid reductoisomerase | **ilvC** | b3774 |  |  | Amino acid metabolism |
| hypothetical protein CRP_126 |  |  |  |  |  |
| acetolactate synthase large subunit | **ilvI** | b0077 |  |  | Amino acid metabolism |
| ribosomal large subunit pseudouridine synthase | **rluD** | b2594 |  |  | Ribosome function, maturation and modification |
| isoleucyl-tRNA synthetase | **ileS** | b0026 |  |  | Aminoacyl-tRNA synthetases |
| hypothetical protein CRP_130 |  |  |  |  |  |
| putative unfunctional GTPase | **obgE (yhbZ)** | b3183 | putative GTPase | GTP-binding domain is lost | Ribosome function, maturation and modification |
| ribosomal protein L27 | **rpmA** | b3185 |  |  | Ribosomal proteins |
| putative unfunctional ribosomal protein L21 | **rplU** | b3186 | hypothetical protein CRP_133 | Identified by syntheny | Ribosomal proteins |
| ribosomal protein L17 | **rplQ** | b3294 | hypothetical protein CRP_134 | Identified by homology. | Ribosomal proteins |
| RNA polymerase alpha subunit | **rpoA** | b3295 |  |  | Transcription |
| ribosomal protein S4 | **rpsD** | b3296 |  |  | Ribosomal proteins |
| ribosomal protein S11 | **rpsK** | b3297 |  |  | Ribosomal proteins |
| ribosomal protein S13 | **rpsM** | b3298 |  |  | Ribosomal proteins |
| ribosomal protein L36 | **rpmJ** | b3299 |  |  | Ribosomal proteins |
| putative ribosomal protein L15 | **rplO** | b3301 |  |  | Ribosomal proteins |
| ribosomal protein S5 | **rpsE** | b3303 |  |  | Ribosomal proteins |
| ribosomal protein L18 | **rplR** | b3304 | hypothetical protein CRP_142 | Identified by homology. | Ribosomal proteins |
| ribosomal protein L6 | **rplF** | b3305 |  |  | Ribosomal proteins |
| putative ribosomal protein S8 | **rpsH** | b3306 | ribosomal protein S8 | N-terminal region very degraded | Ribosomal proteins |
| ribosomal protein S14 | **rpsN** | b3307 |  |  | Ribosomal proteins |
| ribosomal protein L5 | **rplE** | b3308 |  |  | Ribosomal proteins |
| ribosomal protein L14 | **rplN** | b3310 |  |  | Ribosomal proteins |
| ribosomal protein S17 | **rpsQ** | b3311 |  |  | Ribosomal proteins |
| ribosomal protein L16 | **rplP** | b3313 |  |  | Ribosomal proteins |
| ribosomal protein S3 | **rpsC** | b3314 |  |  | Ribosomal proteins |
| putative ribosomal protein L22 | **rplV** | b3315 |  |  | Ribosomal proteins |
| ribosomal protein S19 | **rpsS** | b3316 |  |  | Ribosomal proteins |
| ribosomal protein L2 | **rplB** | b3317 |  |  | Ribosomal proteins |
| ribosomal protein L4 | **rplD** | b3319 |  |  | Ribosomal proteins |
| putative unfunctional ribosomal protein L3 | **rplC** | b3320 | ribosomal protein L3 | Region N-terminal completely lost | Ribosomal proteins |
| ribosomal protein S10 | **rpsJ** | b3321 |  |  | Ribosomal proteins |
| elongation factor Tu | **tuf** | b3980 |  |  | Translation factors |
| elongation factor G | **fusA** | b3340 |  |  | Translation factors |
| ribosomal protein S7 | **rpsG** | b3341 |  |  | Ribosomal proteins |
| ribosomal protein S12 | **rpsL** | b3342 |  |  | Ribosomal proteins |
| RNA polymerase beta' subunit | **rpoC** | b3988 |  |  | Transcription |
| RNA polymerase beta subunit | **rpoB** | b3987 |  |  | Transcription |
| ribosomal protein L7/L12 | **rplL** | b3986 |  |  | Ribosomal proteins |
| putative unfunctional ribosomal protein L10 | **rplJ** | b3985 | hypothetical protein CRP_164 | Identified by syntheny | Ribosomal proteins |
| putative unfunctional ribosomal protein L1 | **rplA** | b3984 | hypothetical protein CRP_165 | Identified by syntheny | Ribosomal proteins |
| ribosomal protein L11 | **rplK** | b3983 |  |  | Ribosomal proteins |
| cytochrome O ubiquinol oxidase subunit IV | **cyoD** | b0429 | putative cytochrome O ubiquinol oxidase subunit IV |  | Energy metabolism |
| cytochrome O ubiquinol oxidase subunit III | **cyoC** | b0430 |  |  | Energy metabolism |
| cytochrome O ubiquinol oxidase subunit I | **cyoB** | b0431 |  |  | Energy metabolism |
| cytochrome O ubiquinol oxidase subunit II | **cyoA** | b0432 |  |  | Energy metabolism |
| putative unfunctional homoserine kinase | **thrB** | b0003 | hypothetical protein CRP_171 | Identified by homology. | Amino acid metabolism |
| branched-chain amino acid aminotransferase |  |  |  |  |  |
| histidyl-tRNA synthetase | **hisH** | b2023 |  |  | Amino acid metabolism |
| hypothetical protein CRP_174 |  |  |  |  |  |
| peptide deformylase | **def** | b3287 |  |  | Protein processing, folding and secretion |
| putative unfunctional argininosuccinate lyase | **argH** | b3960 | hypothetical protein CRP_176 | Identified by homology. Important catalytic residues are lost | Amino acid metabolism |
| diaminopimelate decarboxylase | **lysA** | b2838 |  |  | Amino acid metabolism |
| diaminopimelate epimerase | **dapF** | b3809 |  |  | Amino acid metabolism |
| glycyl-tRNA synthetase alpha subunit | **glyQ** | b3560 |  |  | Aminoacyl-tRNA synthetases |
| hypothetical protein CRP_180 |  |  |  |  |  |
| cold shock protein | **cspE** | b0623 |  |  | Transcription |
| hypothetical protein CRP_182 |  |  |  |  |  |

*^a^* Putative unfunctional genes (in red) present orthologs in γ-proteobacteria, but they have lost essential regions that probably impair function.

*^b^* The orthologous gene in *E. coli* encodes cardiolopin synthase. Only the PDC domain is present in the *C. ruddii* genome.

**Supplementary references**

1. Guenther BD, Sheppard CA, Tran P, Rozen R, Matthews RG, Ludwig ML: **The structure and properties of methylenetetrahydrofolate reductase from Escherichia coli suggest how folate ameliorates human hyperhomocysteinemia**. *Nat Struct Biol* 1999, **6**(4):359-365.

2. Zhang Y, Li X, Spremulli LL: **Role of the conserved aspartate and phenylalanine residues in prokaryotic and mitochondrial elongation factor Ts in guanine nucleotide exchange**. *FEBS Letters* 1996, **391**(3):330-332.

3. Lancaster L, Kiel MC, Kaji A, Noller HF: **Orientation of Ribosome Recycling Factor in the Ribosome from Directed Hydroxyl Radical Probing**. *Cell* 2002, **111**(1):129-140.

4. Ivanova N, Pavlov MY, Bouakaz E, Ehrenberg M, Schiavone LH: **Mapping the interaction of SmpB with ribosomes by footprinting of ribosomal RNA**. *Nucl Acids Res* 2005, **33**(11):3529-3539.

5. Ponting CP, Kerr ID: **A novel family of phospholipase D homologues that includes phospholipid synthases and putative endonucleases: Identification of duplicated repeats and potential active site residues**. *Protein Sci* 1996, **5**(5):914-922.
