# Supplementary material for: The frontier between cell and organelle: genome analysis of Candidatus Carsonella ruddii
Source: BMC Evol Biol. 2007 Oct 1;7:181. doi: 10.1186/1471-2148-7-181 (PMC2175510; doi:10.1186/1471-2148-7-181)
Supplement: Additional file 3 — Schematic representation of amino acid biosynthetic pathways in E. coli (blue arrows), B. aphidicola BAp (green arrows), and C. rudii (red arrows). Only incomplete pathways in C. rudii are shown. Hatched arrows in C. rudii indicate genes that are present in its genome but are greatly degraded. These genes probably have lost their original functionality. Open arrows show activities that have not been found in the genome but could be encoded by some other gene. The metabolites shown in magenta must be provided by the host. [file 1471-2148-7-181-S3.pdf]

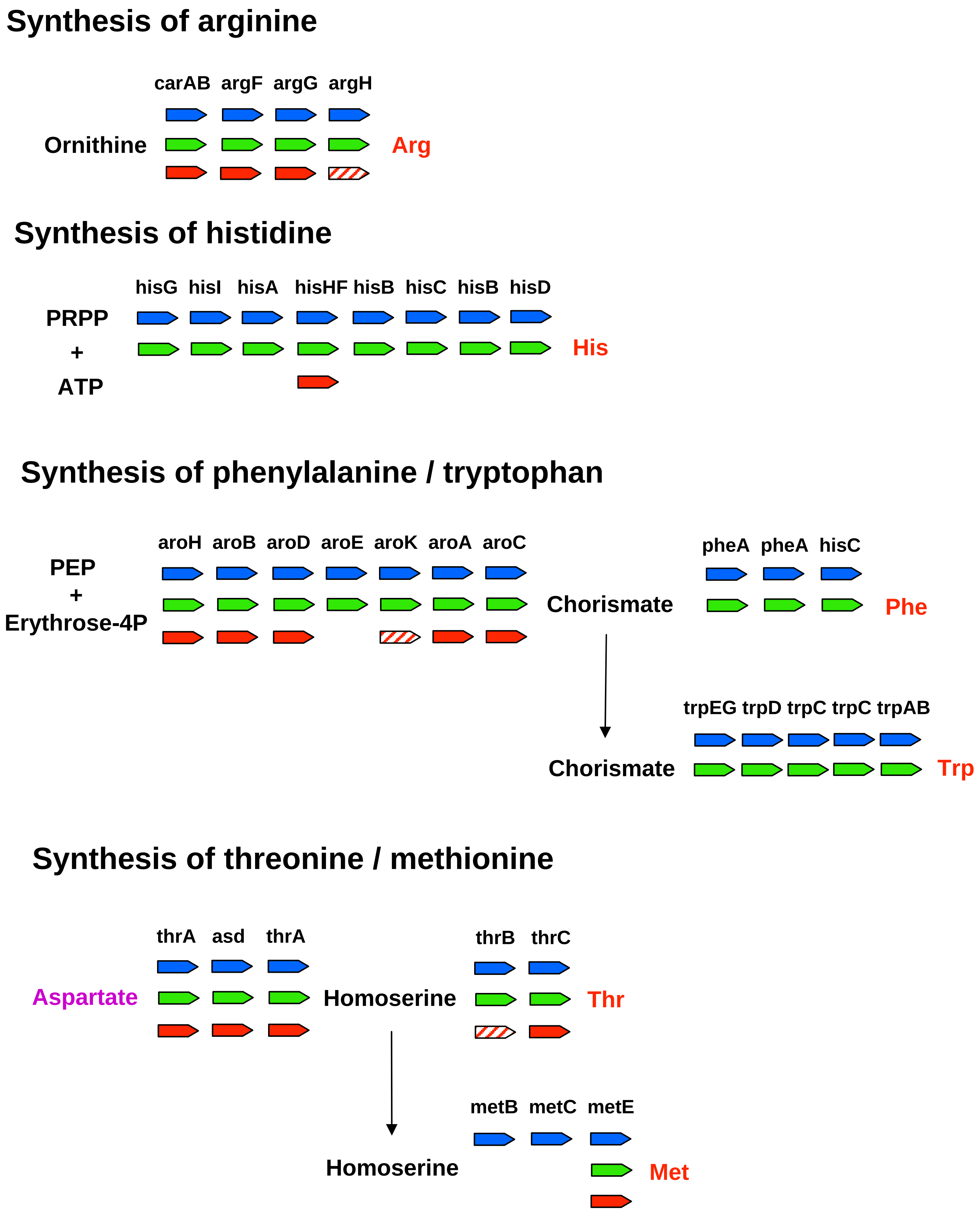
Synthesis of arginine
carAB argF argG argH
Ornithine
Arg
Synthesis of histidine
hisG hisI hisA hisHF hisB hisC hisB hisD
PRPP
+
ATP
His
Synthesis of phenylalanine / tryptophan
aroH aroB aroD aroE aroK aroA aroC
PEP
+
Erythrose-4P
Chorismate
pheA pheA hisC
Phe
trpEG trpD trpC trpC trpAB
Chorismate
Trp
Synthesis of threonine / methionine
thrA asd thrA
Aspartate
Homoserine
thrB thrC
Thr
metB metC metE
Homoserine
Met

Figure S2
